# Towards a Holistic Understanding of the Interactions of Redox-Active Proteins

**DOI:** 10.3390/antiox13111306

**Published:** 2024-10-28

**Authors:** Alexios Vlamis-Gardikas

**Affiliations:** Division of Organic Chemistry, Biochemistry and Natural Products, Department of Chemistry, University of Patras, 26504 Rion, Greece; avlamis@upatras.gr

## 1. Introduction

The deciphering of the organization of molecular life in cells starts with the analysis of their biochemical pathways. This is an ongoing process aimed at the identification of specific chemical reactions and the proteins involved. All organisms rely on mechanisms that provide energy by some type of electron flow, while the reversal of undesired electron flow is the work of antioxidants. A considerable number of proteins participate in the transfer of electrons to and from large or small molecules. Defining the types and characteristics of interactions among redox-active proteins is therefore necessary to understand the biochemistry of life.

How are protein–protein interactions studied? Qualitative aspects may be identified by immunoprecipitation experiments (proximity labeling included), photoaffinity labeling, affinity chromatography using immobilized (mutant) baits, two hybrid screens, and genetic complementation, among others. Equilibrium dialysis, optical spectroscopy, surface plasmon resonance, and isothermal titration calorimetry are some of the methods widely used for calculating quantitative parameters of the interactions. Structural studies by NMR, crystallography, and hydrogen-deuterium exchange mass spectrometry have all been used for determining the exact areas of interaction and contact residues between a protein of interest and its ligands. Of course, these are only some of the methods used. Innovation in the field is continuous (especially in biophysics), while advanced in silico methods are contributing to the topic (e.g., AlphaFold 3) and will do even more in the future with the advancement and innovations in the field of computing science.

## 2. The Delineation of Redox Interactomes Entails Therapeutic Potential

Particularities in electron chains among distant taxonomic groups of organisms have been exploited for the benefit of mankind using xenobiotics to eliminate undesired organisms. The pesticide inhibitors of photosynthesis [1] are one such well-known example. Subtle differences in the enzyme systems of similar functions across different taxa have served as the principle for finding specialized inhibitors. The latter may dissimilarly affect enzymes from different species (e.g., between mammals and bacteria [2]) or within the same taxonomic rank (Gram-positive versus Gram-negative bacteria) [2,3]. Such information may form the basis for therapeutic interventions and is of course not restricted to systems of electron flow (e.g., inhibitors of topoisomerases [4]).

Modern medicine requires that drugs perform predefined tasks ranging from broad-spectrum actions (as seen with many existing compounds) to highly selective aims. These drugs should act on specific organisms, organs, and biochemical reactions, preferably with no side effects. Redox chain reactions entail a high degree of evolutionary conservation regarding the use of thiolates in electron relays. As inhibitors of electron chains may generally target nucleophilic atoms such as thiolates, their inhibitory effect may affect diverse systems, leading to undesirable side effects. Hence, it would be preferable to develop selective inhibitors that do not interact covalently with electrophilic centers but rather block interactions between the unique surfaces with which a protein couple interacts in a particular electron flow. These inhibitory compounds would most likely target contact areas/protein–protein interfaces containing key residues (thermodynamic hot spots) whose contribution to the free energy of the interaction is high. Ongoing approaches have devised specific peptides or peptidomimetics, non-peptide mimetics (stable organic molecules), for the disruption of specific protein–protein interaction [5,6,7]. The use of such inhibitors may thus prove effective in inhibiting selected interactions between any couple of proteins in an existing electron chain. This seems especially relevant for multipartner proteins (as proposed for HsTrx2 in [8]): selective inhibitors could block the interaction of specific protein pathways of a multipartner protein by blocking selected contact areas (protein–protein interphases) without affecting its other contacts. To implement these strategies, the specific interacting partners and their precise mode of interaction must first be identified.

## 3. Presentation of the Special Issue

Has the current Special Issue [8] addressed some of these considerations? In the article “Oxidation of Arabidopsis thaliana COX19 Using the Combined Action of ERV1 and Glutathione” [8], biochemical approaches were used to show fine differences between plants and yeast in a mechanism that imports proteins to mitochondria. If one were to seek a very fine point of differentiation between yeast and plants (perhaps for the development of fungicides), this transporter could be one such. The article entitled “Functional Diversity of Homologous Oxidoreductases—Tuning of Substrate Specificity by a FAD-Stacking Residue for Iron Acquisition and Flavodoxin Reduction” [8] focused on the role of a key residue of *Bacillus cereus,* FNR2, that may determine the interaction of FNRs with endogenous substrates such as the Fld-like protein NrdI. Here, differences in the interaction of a very similar group of enzymes with a substrate are highlighted by X-ray crystallography. Could these differences form a basis for the design of possible inhibitors for the interaction, and what would be the practical consequences that might arise from such developments? In “Disruption of Bacterial Thiol-Dependent Redox Homeostasis by Magnolol and Honokiol as an Antibacterial Strategy” [8], the electron flow within the thioredoxin (Trx) system of *Staphylococcus aureus* is inhibited by compounds from traditional Chinese medicine. The therapeutic potential and selectivity of these compounds for staphylococcal infections are demonstrated in a mouse model. One could envisage that future experiments on the identification of the contact areas of these compounds with the components of the Trx system could provide insights for surfaces with potential pharmacological significance. In “The Functional Relationship between NADPH Thioredoxin Reductase C, 2-Cys Peroxiredoxins, and m-Type Thioredoxins in the Regulation of Calvin–Benson Cycle and Malate-Valve Enzymes in Arabidopsis” [8], the role of m-type Trxs in the light-dependent regulation of biosynthetic enzymes and the malate valve under the control of the NADPH Trx reductase C and the 2-Cys-peroxiredoxin system are explored. This contribution adds to the understanding of how Prxs fine-regulate photosynthesis in plants. Could this knowledge be applied to optimize photosynthesis in different plant species or have some application in the selective treatment/elimination of undesirable plants groups? The review article “Deciphering the Role of Selenoprotein M” [8] presents the most recent advances on the interacting partners and respective roles of the protein in health and disease. In the other review paper “SLC7A11 as a Gateway of Metabolic Perturbation and Ferroptosis Vulnerability in Cancer” [8], the relationship between the transmembrane protein SLC7A11 (which transports extracellular cystine into cells for cysteine production and GSH biosynthesis) and the sensitivity of cancer cells to ferroptosis is discussed. Could SLC7A11 become a pharmacological target for anti-cancer drugs? In “Distinct or Overlapping Areas of Mitochondrial Thioredoxin 2 May Be Used for Its Covalent and Strong Non-Covalent Interactions with Protein Ligands” [8], the preferred surface areas of mitochondrial Trx2 (HsTrx2) for covalent and non-covalent interactions are presented by utilizing the existing interactome of HsTrx2 and in silico docking approaches. HsTrx2 appeared as a protein belonging to a “Multi hub” system [9] with different binding areas for its various protein ligands. The principle for the design of selective inhibitors for certain areas—interactors of HsTrx2 was hence proposed. Considering the overall composition and presentation of the topics, the Special Issue has only partially addressed its title. This was not unexpected given the enormousness of the subject. However, the shortcomings highlight the need for more research on the topic.

## 4. The Analysis of Redox Interactomes May Require International Collaboration

The possibility for therapeutic intervention in protein–protein interaction relays such as those of electron flows is obvious. Specific links in the chains of protein–protein interaction could be disrupted by selective inhibitors that would hinder the chosen interaction and prove of therapeutic potential. To this end, the protein interactomes of many redox-active proteins remain to be fully elucidated and the individual interactions analyzed. Global projects such as the Structural Genomics Consortium [10] concerning the analysis of protein structure by X-ray crystallography and NMR have been quite successful in providing the international community with structures for academic and commercial applications. A reasonable step would be the development of future multicenter initiatives on identifying first the interactomes of redox proteins followed by the detailed analysis of the detected protein–protein interactions. A key concern is how to standardize the methods and protocols for the analyses, especially the detection of interactomes. This requires the consideration of the specific circumstances (phase of growth, environmental conditions, cell type) under which proteomes are composed. Even then, the quest for only covalent interactions for a given protein could limit the number of detected ligands.

## 5. The Ignored Non-Covalent Interactomes—Future Prospects

A common approach for determining the covalent partners of redox-active proteins with active sites composed of vicinal thiols of the CxxC type entails the use of a monothiol bait of the CxxS type (C is cysteine, S is serine). The elimination of the second resolving cysteine (which may also be mutated to alanine) of the active site may trap substrates involved in thiol-disulfide exchange. However, there is some uncertainty regarding the specificity of this method derived from the conditions applied to stimulate the nucleophilic attack of the artificial monothiol active site cysteine (bait) to the target dithiol. It is possible that the attack may not accurately reflect the in vivo situation, leading to a significant number of false positives. This issue may be exacerbated by the high sensitivity of contemporary tools of mass spectrometry used to analyze the interacting proteins. While concerns about the non-specificity of this method are not unreasonable, an emerging concept in cellular life is that the assignment of a single function to a given protein species does not hold for an increasing number of proteins, including the Trx from *E. coli* [11]. Also, in *Salmonella*, endogenous Trx interacted with many response regulator proteins of the mammalian host [12]. It is thus quite possible that other redox-active proteins may interact with more than one protein ligand to perform unrelated functions, a phenomenon known as protein moonlighting. In the case of *E. coli* Trx, this has been shown by the large number of proteins identified by the monothiol trapping approach (e.g., [13]). Such multipartner proteins exhibit varying affinities for their respective ligands. Skeptics may reasonably consider some of the observed interactions as false positives, and this could be the case for a number of them. Obviously, unless each pair of detected interactions is examined thoroughly, the reported interactions, particularly for the highly expressed ligands, may remain in doubt. However, even this questioned richness of transient covalent interactions at the active site is likely an underrepresentation of the total interactome of a redox-active protein, as it does not consider the occurring non-covalent interactions. Redox-active proteins may engage with additional protein ligands in a non-covalent manner. One of the earliest examples of a strong non-covalent interaction between a redox-active protein and another protein is the complex of T7 DNA polymerase formed by the gene 5 protein of phage T7 with reduced Trx1 from *E. coli* [14]. This complex is so robust that it can only be dissociated by basic or acidic conditions. In the case of HsTrx2, nearly half of the interacting protein ligands were detected in acid eluates, indicating the abundance of strong non-covalent interactions occurring between HsTrx2 and its protein ligands [14]. Despite this, studies examining the non-covalent interaction of redox-active proteins are relatively scarce; the topic appears somewhat ignored. These strong interactions could, in principle, modify electron flow or even lead to the discovery of novel functions. Given the method used for their detection (acidic elutions preceded by salt washes), the reported [15] strong interactions are most likely not false positives but rather genuine non-covalent interactions. Furthermore, to our knowledge, the weaker non-covalent interactions of redox proteins have not been examined at all. These overlooked interactions may be significant for cellular function: although association constants may not appear optimal, the plentifulness of interacting pairs could force equilibria towards the formation of seemingly unfavored complexes. Moreover, affinity experiments concerning non-covalent interactions could reveal the role of proteins with Trx-like folds and similar active sites that have not shown significant in vitro activity in standard assays (e.g., insulin precipitation by DTT or reduction assays). Clearly, further studies on the transfer of electrons by protein–protein relays and the greater subject of protein–protein interaction would certainly address basic academic questions and, in the long term, pave the way for the design of novel highly selective drugs. To this aim, an internationally organized network of different laboratories with standardized experimental approaches could be the way to proceed further.
